# A Multiplex Assay to Measure RNA Transcripts of Prostate Cancer in Urine

**DOI:** 10.1371/journal.pone.0045656

**Published:** 2012-09-20

**Authors:** Sue-Ing Quek, Melissa E. Ho, Michelle A. Loprieno, William J. Ellis, Nathan Elliott, Alvin Y. Liu

**Affiliations:** 1 Department of Urology, University of Washington, Seattle, Washington, United States of America; 2 Institute for Stem Cell and Regenerative Medicine, University of Washington, Seattle, Washington, United States of America; 3 nanoString Technologies, Seattle, Washington, United States of America; The Chinese University of Hong Kong, Hong Kong

## Abstract

The serum prostate-specific antigen (PSA) test has a high false positive rate. As a single marker, PSA provides limited diagnostic information. A multi-marker test capable of detecting not only tumors but also the potentially lethal ones provides an unmet clinical need. Using the nanoString nCounter gene expression system, a 20-gene multiplex test was developed based on digital gene counting of RNA transcripts in urine as a means to detect prostate cancer. In this test, voided urine is centrifuged to pellet cells and the purified RNA is amplified for hybridization to preselected probesets. Amplification of test cell line RNA appeared not to introduce significant bias, and the counts matched well with gene abundance levels as measured by DNA microarrays. For data analysis, the individual counts were compared to that of β2 microglobulin, a housekeeping gene. Urine samples of 5 pre-operative cases and 2 non-cancer were analyzed. Pathology information was then retrieved. Signals for a majority of the genes were low for non-cancer and low Gleason scores, and 6/6 known prostate cancer markers were positive in the cases. One case of Gleason 4+5 showed, in contrast, strong signals for all cancer-associated markers, including CD24. One non-cancer also showed signals for all 6 cancer markers, and this man might harbor an undiagnosed cancer. This multiplex test assaying a natural waste product can potentially be used for screening, early cancer detection and patient stratification. Diagnostic information is gained from the RNA signatures that are associated with cell types of prostate tumors.

## Introduction

With an aging population, the number of prostate cancer cases will increase dramatically in the next two decades. The serum PSA test is used to monitor men for prostate cancer and many tumors have been detected early through its use. However, almost ¾ men with abnormal PSA turn out to be negative for cancer upon biopsy [1]. Thus, many of the biopsies performed are unnecessary. Also, some cancer cases are undetected by PSA. While PSA is abundantly produced by the prostate it is not cancer-specific although molecular forms of PSA such as [-2]proPSA appear to show better performance [2]. Other markers are needed for definitive cancer diagnosis. In addition, markers are needed to differentiate prostate cancers that could be lethal from those that are not. A multi-marker test is required. The nanoString nCounter technology can directly measure multiple RNA transcripts in biospecimens, and the readout is digital in gene counts [3]. In the nCounter system, two oligonucleotide probes are designed for each transcript target: one conjugated to biotin and the other color-coded with dye molecule combinations. After solution hybridization, the captured transcripts are immobilized on a streptavidin-coated surface, aligned in an electrical field, and identified by their individual color codes. The codes are counted with a microscope/camera device and tabulated in Excel. For technical reasons, the maximum number of genes that can be analyzed per run is just below 800. The technology has a sampling efficiency of ∼1% (i.e., 1,800 molecules produced 25 counts), a signal range from ∼25 to >50,000 counts, and good reproducibility [3].

Our objective is to develop this nanoString technology for prostate cancer detection through the analysis of voided urine since the prostate is drained by the urinary tract. Various cell types including diseased ones might be shed or released from organs along this tract. For the nCounter probesets, informative prostate cancer genes were identified through transcriptomic analysis of cancer epithelial and cancer-associated stromal cell types against their respective non-cancer counterpart. To date, the relevant cell types included Gleason pattern 3 CD26^+^ cancer cells, Gleason pattern 4 CD26^+^ cancer cells, CD90^+^ cancer-associated stromal cells to contrast with CD26^+^ luminal cells and CD49a^+^ stromal cells [4], [5]. Selected marker genes were incorporated into the nanoString prostate cancer probe library to produce the nCounter CodeSet. Urine collection represents the least invasive route of obtaining test material, and being a waste product, multiple samples can be conveniently collected without risk or stress to the patients. Since cell types of tumors have distinct gene expression, their gene signature should not be present in healthy non-cancer. There is also the possibility that due to the abnormal histoarchitecture of tumors, cancers as well as cancer-associated cells are more likely to be released into the urine than cells in normal, especially in cases where the extracellular matrix is being destroyed by tumor-derived metalloproteinases.

The Gen-Probe PCA3 urine test is one currently based on the presence of cancer cells in urine for disease diagnosis [6]. It measures a non-coding RNA (PCA3) through target capture and amplification, chemiluminescent probe detection with readout in relative light units. A multicenter evaluation of the test showed acceptable analytical performance and a correct subject classification (cancer *vs*. biopsy negative) of just under 70% [7]. As a prognostic marker, PCA3 showed no significant link to Gleason score, tumor volume, stage, based on 70 cases [8]. Another study reported a link to these clinicopathological features [9]. Additional markers can be included using whole transcriptome amplification followed by polymerase chain reaction (PCR) [10]. Three other markers (PSMA, HPN, GALNT3) plus PCA3, for example, could distinguish cancer from benign at 100% in one report [11]. Multiplex PCR, however, is not a robust technology and cumbersome to perform in a high throughput setting. The nCounter technology not only provides multi-marker analysis capability but also ease-of-use. Payton *et al*. [12] reported the application of nCounter to measure RNA abundance of acute myeloid leukemia. Our development of a multiplex (PCA3 plus other markers) test is motivated by similar tests offered to breast cancer patients for stratification [13].

## Materials and Methods

### Ethics Statement

This study was approved by the University of Washington Institutional Review Board. Voided urine specimens and excess tissue specimens from surgeries were collected from donors with written consent.

### Probeset Selection

The nCounter probesets were designed and synthesized by nanoString Technologies [3]. According to the nanoString CodeSet design, each probe pair was made complementary to a 100-base region of the target RNA. The capture probe was conjugated to biotin for immobilization onto a streptavidin-coated plate after hybridization, and the reporter probe to a color-coded tag for signal detection. Twenty gene markers were selected (Table 1). Of these, six were genes reported by multiple groups to show increased expression in prostate cancer: AGR2 (anterior gradient 2) [14], AMACR (α-methylacyl-CoA-racemase) [15], CRISP3 (cysteine-rich secretory protein 3) [16], HPN (hepsin) [17], PCA3 [18], and ERG (*v*-ets erythroblastosis virus E26 oncogene homolog, activated as a result of gene fusion to the androgen-regulated *TMPRSS2*) [19]. B2M (β2 microglobulin) was used as control for RNA quality and quantity. BRE (brain and reproductive organ-expressed TNFRSF1A modulator), IL24, CCL3 [chemokine (C-C motif) ligand 3, macrophage inflammatory protein 1 subunit MIP1α] were detected as up-regulated genes in prostate cancer cells [4]. ACPP (prostatic acid phosphatase), AZGP1 (zinc α2-glycoprotein 1), KLK2 [(hK2) and KLK3], ANPEP (alanyl aminopeptidase, CD13), CD24, CD9, DPP4 (dipeptidyl-peptidase 4, CD26), MME (membrane metallo-endopeptidase, CD10) were included as prostate luminal cell genes with differential expression between cancer and non-cancer [20]. CD90v (CD90 variant BAD92446, and CD90/THY1) was included for cancer-associated stromal cells [5], [21], and UPK3A (uroplakin 3A) for bladder cells [22].

**Table 1:** nCounter probesets.

	CODESET DETAILS		
	Customer Identifier	Accession	Targeted Region
1	AMACR	NM_203382.1	568–668
2	BRE	NM_004899.3	230–330
3	ERG	NM_182918.2	90–190
4	AGR2	NM_006408.2	1365–1465
5	IL24	NM_006850.2	651–751
6	CRISP3	NM_006061.1	700–800
7	HPN	NM_182983.1	1870–1970
8	PCA3	NR_015342.1	2645–2745
9	MME	NM_000902.2	5059–5159
10	ANPEP	NM_001150.1	2670–2770
11	DPP4	NM_001935.3	2700–2800
12	CD90 variant - BAD92446	AB209209.1	765–865
13	CCL3	NM_002983.2	681–781
14	UPK3A	NM_006953.2	715–815
15	CD9	NM_001769.2	405–505
16	CD24	NM_013230.2	95–195
17	ACPP	NM_001099.2	930–1030
18	AZGP1	NM_001185.2	123–223
19	KLK2	NM_005551.3	1820–1920
20	B2M	NM_004048.2	25–125

Probe sequences are selected from gene entries identified by the accession numbers listed.

### Prostate Cancer Cell Lines

The prostate cancer cell lines PC3, C4-2, and C4-2B were maintained in RPMI1640 media supplemented with 10% fetal bovine serum. The transcriptomes of PC3 and C4-2 were reported previously [23]. C4-2B was derived from C4-2 for growth in (mouse) bone [24]. PC3 and C4-2 were obtained from the American Type Culture Collection (Manassas, VA). C4-2B [25] was kindly provided by Dr. Robert Vessella in the Urology department.

**Figure 1:**
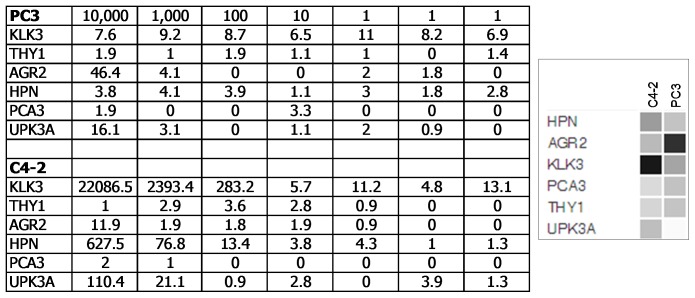
Digital gene counts. The table shows the normalized gene counts in a dilution series of 10,000, 1,000, 100, 10, 1 (3x) cells of PC3 *vs*. C4-2. The genes are listed in the first column. The dataset query display for expression levels of these genes in C4-2 and PC3 is shown on the right.

### Urine Collection and Processing

Voided urine specimens were collected from donors. No special pre-collection preparation was required of the patients. Within 2 h, the urine (20 to 100 ml) was poured from collection jars into 50-ml sterile conical tubes, and centrifuged at 1,500 rpm for 5 min. The supernatant was saved (for protein analysis); the pellet was resuspended in residual urine and transferred to 1.5-ml Eppendorf tubes for <1 min in a microcentrifuge at maximum speed. No more than 10 µl RLT buffer (Qiagen, Valencia, CA) containing 1% β-mercaptoethanol was added. The cell lysates were stored at −80°C until analysis (nCounter hybridization or RNA extraction and amplification).

**Figure 2:**
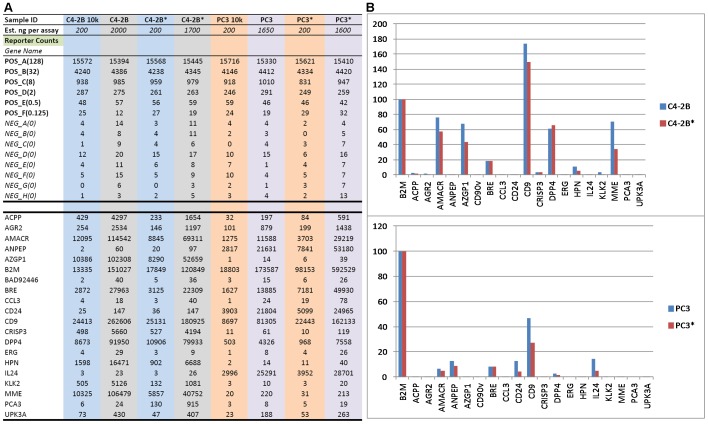
RNA amplification. A. Shown is Excel format of nCounter data from unamplified *vs.* amplified (marked by *) RNA of C4-2B and PC3 cells. POS and NEG are control probesets. **B.** Signal counts obtained from unamplified *vs*. amplified (marked by *) are compared in histogram display. Gene count for B2M is set at 100 and all other gene counts are compared to that of B2M from the same sample. Note genes with absent expression were not amplified.

### RNA Extraction and Amplification

Urine RNA was prepared with RNAqueous Micro (Ambion, Life Technologies, Carlsbad, CA). Briefly, 90 µl of Lysis Solution was added to the 10 µl cell lysates with vigorous mixing. Fifty µl ethanol was then added, and the mixture was loaded onto a Micro-Filter Cartridge Assembly. The cartridge was washed, and RNA was eluted in 2×8 µl of preheated Elution Solution. Depending on the RNA concentration, 3–5 µl of the eluted RNA (30–50 ng) was used for RNA amplification using RiboMultiplier Sense-RNA Amplification (Epicentre, Illumina, Madison, WI). First-strand cDNA synthesis, terminal oligomer tagging, second-strand cDNA synthesis, *in vitro* transcription of sense RNA and DNase I digestion were performed following the manufacturer’s protocol in a Thermocycler. Amplified RNA was purified using RNeasy Mini (Qiagen) and resuspended in 30 µl of RNase-free water. RNA from cell lines was prepared with RNeasy Mini.

### NanoString nCounter Hybridization and Presentation of Gene Counts

Probe hybridization was carried out with 4–5 µl of cell lysates or 100–2,000 ng RNA for 18 h in an automated nCounter machine. All genes on the nCounter CodeSet were analyzed simultaneously in true multiplex fashion. Raw counts were first normalized using the mean of six internal spike-in positive control probes for all samples to account for systematic differences between assays. These control-normalized counts were then compared to the expression level of B2M (set at 100 for convenience). For data presentation, each gene count was indicated in relation to that of B2M by multiplying a factor of 100/(B2M gene count). The nCounter data were obtained before pathology of the cancer was retrieved, i.e., blinded to Gleason score and stage of the tumors. These pathology features were ascribed to the case numbers in the results section.

**Figure 3:**
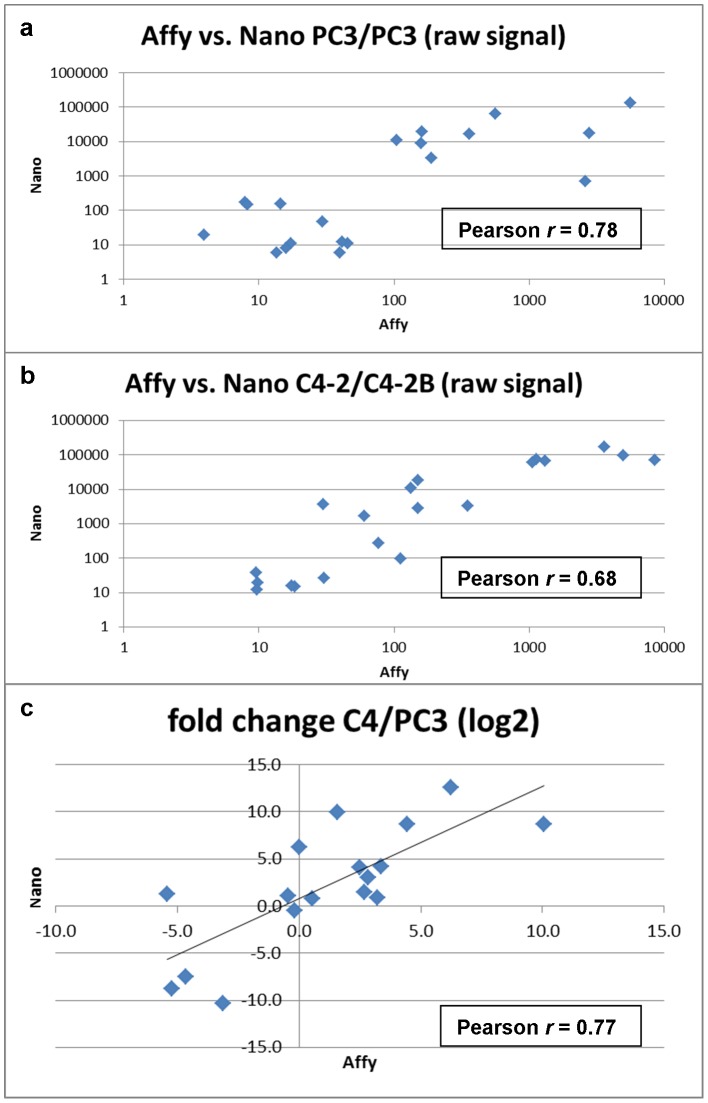
Correspondence between nCounter and DNA microarray analysis. Comparison of the raw signal values obtained for (a) PC3 and (b) C4-2/C4-2B cells are shown. The bottom panel (c) shows the comparison of fold-change calculated from the obtained data values.

### Immunohistochemistry

Serial 5-µm frozen sections of tumors were processed for immunostaining with antibodies to cluster designation (CD) cell surface antigens or other markers as described [20]. Monoclonal antibody to CD24 (clone ML5, 1∶50) was obtained from BD PharMingen (San Diego, CA), monoclonal antibody to CCL3 (clone 4E7, 1∶50) was from Abnova (Taiwan), and antibody to AGR2 (clone P1G4, 1∶50) was produced in our lab [26]. The immunostained sections were imaged with Olympus BX41 (Melville, NY) equipped with MicroFire digital camera (Optronics, Goleta, CA). Images were processed with Photoshop CS (Adobe Systems, San Jose, CA).

**Figure 4:**
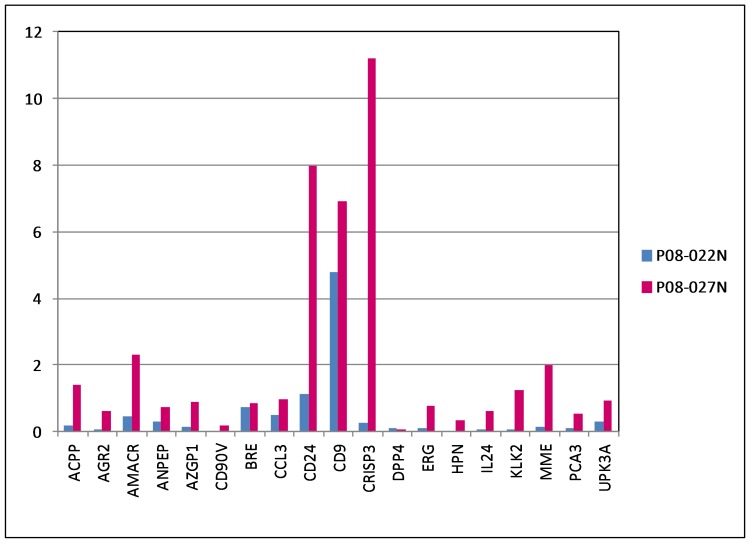
Urine RNA signatures of non-cancer. The histogram data display shows the signal counts for P08-022N (blue) and P08-027N (magenta). The high counts of B2M were excluded for data display. P08-022N shows no signals for the cancer markers. In contrast, P08-027N shows counts for these markers.

## Results

### Limit of Detection

RNA from a dilution series of prostate cancer lines C4-2 and PC3 was prepared, and hybridized to a probe library of six selected genes: KLK3, CD90/THY1, AGR2, HPN, PCA3, UPK3A. The raw counts were normalized, and the data was analyzed by nanoString statistical software tools. The background counts were found to be <15 and the sensitivity limit was ∼20–30 cells based on an abundantly expressed gene like KLK3 in C4-2 (Fig. 1). The counts were in good agreement with cell numbers: 22,100 KLK3 counts for 10,000 cells; 2,400 counts for 1,000; 290 counts for 100. On a per cell basis, C4-2 showed a signature of 2.21 KLK3, 0.06 HPN, 0.01 UPK3A, 0 THY1, 0 AGR2, 0 PCA3, while PC3 showed a signature of 0 KLK3, 0 HPN, 0 UPK3A, 0 THY1, 0.005 AGR2, 0 PCA3. The signature for C4-2 could also be expressed in ratios to the smallest count number: 220 KLK3, 6 HPN, 1 UPK3A. The expression matched to that obtained by DNA microarray analysis.

**Figure 5:**
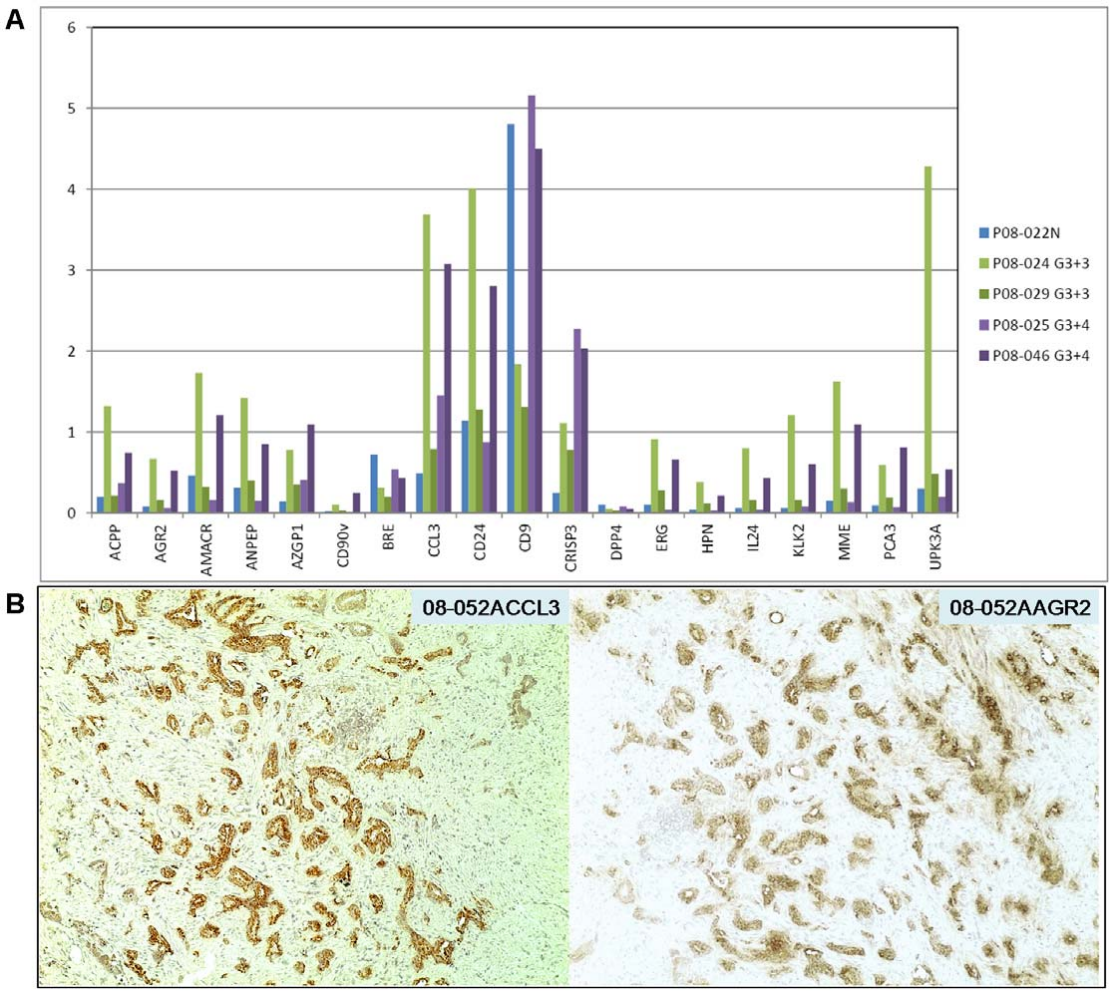
Urine RNA signatures of cancer. A. The signal counts for P08-024pre (Gleason 3+3), P08-029pre (Gleason 3+3, T2c), P08-025pre (Gleason 3+4), and P08-046pre (Gleason 3+4, T2c) are compared to those of P08-022N. Note not only the cancer markers but also the prostate luminal markers produced signals (cancer cells also produce these markers). **B.** Two serial sections of tumor specimen 08-052A were stained for CCL3 and AGR2 expression. Magnification is 100x.

### Gene Expression Profile was Preserved with RNA Amplification

To evaluate RNA amplification, PC3 and C4-2B RNA were amplified and the resultant products hybridized to the nCounter 20-gene CodeSet: ACPP, AGR2, AMACR, ANPEP, AZGP1, B2M, CD90v, BRE, CCL3, CD24, CD9, CRISP3, DPP4, ERG, HPN, IL24, KLK2, MME, PCA3, UPK3A. Fig. 2A shows the RNA count data obtained in Excel format. The signal counts (from ∼2,000 ng input RNA) between unamplified and amplified material were comparatively equivalent for these markers (Fig. 2B). The relative abundance was also maintained. A notable bias was not seen for any transcript. For example, 2,000 ng C4-2B RNA produced 151,027 counts for B2M, 200 ng produced 13,335 counts, and 1,700 ng amplified RNA produced 120,849 counts; for AMACR, the same RNA amounts produced 114,542 counts, 12,095 counts, and 69,311 counts respectively. The most significant result was that non-expressed genes (background counts <25-30) were not detected, for example, the stromal cell gene CD90v: 40 counts, 2 counts, and 36 counts, and IL24∶23 counts, 3 counts, 26 counts respectively for the three amounts of RNA.

**Figure 6:**
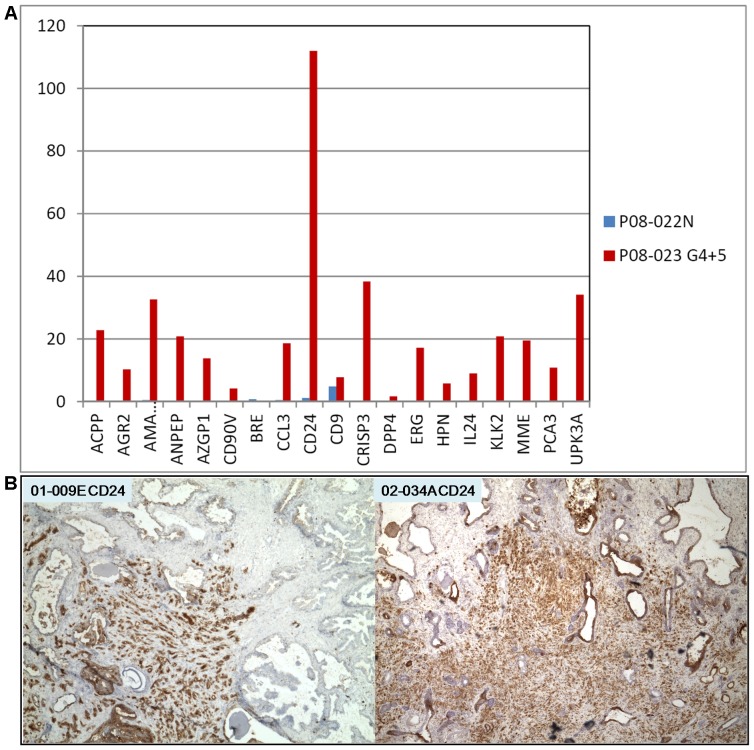
Urine RNA signature of Gleason 4+5. A. The data display shows the high signal counts obtained, especially that of CD24. **B.** CD24 immunohistochemistry shows strongly stained cancer cells in tumor specimens 01-009E and 02-034A. Magnification is 40x.

### Concordance between nCounter and DNA Microarray Profiling

PC3 *vs*. PC3 with no expected difference and C4-2B *vs*. C4-2 with a few differences were purposely chosen for comparison. For PC3, a strong concordance (Pearson *r* = 0.78, Fig. 3) in expression levels between gene counts and array signal values was found. A lower correlation (*r* = 0.68) between C4-2B and C4-2 could be attributed to the few differentially expressed genes between the two, e.g., C4-2B was positive for AGR2 and C4-2 not. Overall, the expression fold difference of the 20 genes in PC3 *vs*. C4-2B/C4-2 showed a correlation of *r* = 0.77. Thus, nCounter could produce an accurate profile of expressed genes and their abundance in particular cell types.

### Urine RNA Signature of Non-cancer

The first cohort of voided urine samples were collected from lab volunteers. The experimental data (not shown) showed that 1) sample preparation was adequate in that there was no significant RNA degradation as indicated by the signals of spiked-in nanoString controls in the samples; 2) larger urine volume did not necessarily yield more cells; 3) signals were generally low without RNA amplification; 4) blood in urine produced spurious readings. In addition, placing prostate cancer cells PC3 and C4-2 in urine for 6 h produced no significant diminution in signal counts (data not shown).

Using RNA amplification, a second cohort of specimens [obtained without digital rectal exam (DRE)] were tested: P08-022N, P08-027N from men with no prior diagnosis of cancer, who visited UW’s Prostate Cancer Awareness; P08-023, P08-024, P08-025, P08-029, P08-046 from pre-op(erative) cancer patients. Using a histogram format for data display, all signal counts for the selected prostate cancer genes were at background for P08-022N: AGR2 = 0.08 [(8 counts, relative to B2M = 100 (10,000 counts)], AMACR = 0.46, CD90v = 0.02, CRISP3 = 0.25, ERG = 0.1, HPN = 0.04, PCA3 = 0.09 (Fig. 4, data entries in blue). There was also little representation of prostate (secretory) cells in the urine as inferred from ACPP = 0.2, AZGP1 = 0.14, KLK2 = 0.06.

**Figure 7:**
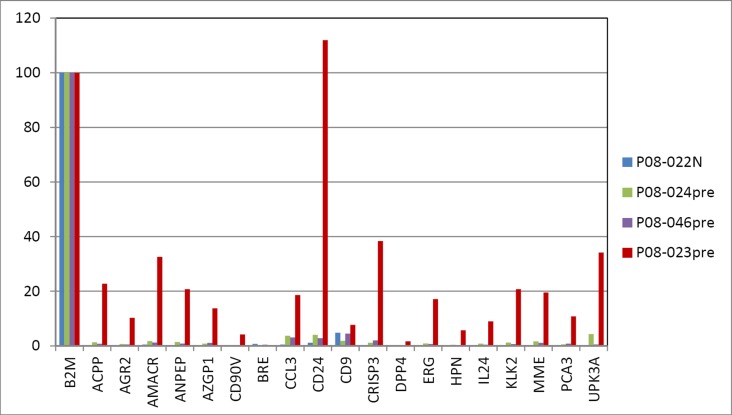
Prostate cancer urine signatures. This composite data display illustrates the difference between Gleason scores (counts of B2M are included).

### A Suspicious N Case

P08-027N (age 54), in contrast, displayed a cancer-like signature: AGR2 = 0.61 (61 counts), AMACR = 2.33, CRISP = 11.21, ERG = 0.76, HPN = 0.34, PCA3 = 0.53 (Fig. 4, data entries in magenta), compared to the non-cancer signature of P08-022N. With 6/6 known prostate cancer markers scoring above background, a good likelihood that the donor was harboring an undiagnosed tumor. In addition, the cancer up-regulated CD24 was at 7.98. There was also higher counts for the prostate genes: ACPP = 1.4, AZGP1 = 0.91, KLK2 = 1.25.

### Signatures for Lower Gleason Cases

The count signals obtained for P08-024pre (Gleason 3+3; PSA = 9.3), P08-029pre (Gleason 3+3; T2c; PSA = 3.6; tumor volume = 0.6 cc), P08-025pre (Gleason 3+4; T2c; PSA = 7; tumor volume = 5 cc), and P08-046pre (Gleason 3+4; T2c; PSA = 26; tumor volume = 5 cc) in comparison to those of non-cancer P08-022N are shown in Fig. 5A. P08-024pre showed AGR2 = 0.67, AMACR = 1.73, CRISP3 = 1.11, ERG = 0.91, HPN = 0.38, PCA3 = 0.59 and P08-046pre showed AGR2 = 0.52, AMACR = 1.21, CRISP3 = 2.03, ERG = 0.66, HPN = 0.21, PCA3 = 0.81. These gene counts for the six cancer markers were >3-fold above the background counts in non-cancer P08-022N. IL24 (0.80 and 0.43, respectively) was found up-regulated in cancer [4]. The increase in CCL3 (3.69 and 3.08, respectively) might indicate the presence of G4 tumor cells as CCL3 was one of the genes upregulated in G4 *vs*. G3 tumor cells [4]. Fig. 5B shows Gleason pattern 4 tumor cells staining for CCL3 (and AGR2) in tissue specimen 08-052A. These gene signals could also be contributed by leukocytes. P08-029pre, which came from a small tumor volume of 0.6 cc, showed marginal increase (2- to 3-fold above background) for these genes except for AMACR: AGR2 = 0.16, AMACR = 0.32, CRISP3 = 0.78, ERG = 0.28, HPN = 0.12, PCA3 = 0.19. On the other hand, P08-025pre showed low counts for these genes except for CRISP3: AGR2 = 0.06, AMACR = 0.16, CRISP3 = 2.27, ERG = 0.04, HPN = 0.03, PCA3 = 0.07.

### Signature of a High Gleason Case

The signal counts for P08-023pre (Gleason 4+5; PSA = 5) were markedly more pronounced (>70-fold above background) compared to N: AGR2 = 10.26, AMACR = 32.61, CRISP = 38.35, ERG = 17.15, HPN = 5.74, PCA3 = 10.8 (Fig. 6A). In particular, a very high CD24 count (111.95) was obtained. A large number of prostate tumors show increased CD24 staining [20], and strongly stained Gleason pattern 5 cells can be seen in Fig. 6B for banked tumor specimens 01-009E and 02-034A. In addition, CD90v = 4.19 for cancer-associated stromal cells was detected. It would appear that in this case, more cancer cells and other cell types were released into the urine. This could be attributed to the loss of histoarchitectural integrity in advanced diseases (which could also lead to higher serum PSA levels). A composite data display in Fig. 7 shows the signal difference between this high-grade cancer and the others.

## Discussion

Using the nanoString technology, we have developed a multi-marker urine test that can potentially be applied for screening, detection and patient stratification of prostate cancer. The RNA signatures obtained from our randomly selected first cohort of clinical urine samples were promising in that they could detect cancer in four out of five pre-op specimens based on the six known prostate cancer markers including PCA3, the current urine-based marker. More importantly, P08-023pre from a patient with high-grade disease showed high RNA counts for many markers. With regard to the suspicious P08-027N case, further testing could not be done as the whereabouts of this one-time donor is unknown.

The PCA3 urine test shows that prostate cancer cells can be found in urine. Unlike the PCA3 test, our urine testing did not require an attentive DRE, although DRE could enhance signals by mechanical means of inducing more cell release from the gland [7]. Thus, the nCounter test could be developed into a non-invasive cancer screening tool. Toward this end, testing of a large cohort of men drawn from the population at large who have no diagnosis of cancer is of critical importance as the baseline expression of the selected prostate cancer markers in urine is unknown.

Many of the prostate cancer markers are also expressed by non-prostatic cell types, for example, AMACR in kidney tubule cells [27], CRISP3 in the secretory epithelium of male genital tract [28], ERG mRNA and protein in endothelial cells (although ERG fusion is specific to prostate cancer) [29], HPN in epididymis and seminal vesicle (The Human Protein Atlas, http://www.proteinatlas.org), AGR2 in the urothelium and kidney tubule cells (The Human Protein Atlas and unpublished data). PCA3 being a noncoding transcript, its expression has not been extensively surveyed by immunohistochemistry as the others. Although the gene signatures of all forms of urogenital cancer/diseases are largely unknown, the six known prostate cancer genes of AGR2, AMACR, CRISP3, ERG, HPN, and PCA3 are at or below background, for example, in bladder cancer [30]. It is likely that each type of urogenital malignancy/disease would give rise to a unique gene signature. The present nCounter codeset is designed specifically for prostate cancer based on the information available to date. Post-op urine (with matched pre-op) is informative to see if all the cancer markers (plus prostate-specific ones) would disappear after surgery. For patients treated by focused radiation, the cancer markers but not ACPP, AZGP1, KLK3 would be lost if the tumor is successfully ablated. Additional questions include: what is the day-to-day variation, if any, in the signature; does the signature have an association with age like serum PSA; what is the number of normal *vs*. tumor cells that are shed into urine.

The nCounter probeset can be expanded to include several more common genes (beside B2M) for gene count comparison purposes, and other markers for detection of specific cell types such as PTPRC/CD45 for white blood cells, PECAM1/CD31 for endothelial cells, NCAM1/CD56, B3GAT1/CD57, NGFR/CD271 for neural elements, UMOD (uromodulin) for kidney cells (and UPK3A for bladder cells). Probe design is not perfect, and sometimes new probesets might be required if the first ones performed poorly, e.g., DPP4/CD26 in the current CodeSet. Both luminal and cancer cells are strongly immunostained for CD26, but the signal counts for DPP4 were generally low. For prostate cancer specificity, men with negative biopsies, benign prostatic hyperplasia, prostatitis, as well as men with bladder and kidney cancer are tested. Specificity is increased by the multiplex format of nCounter. The selected markers are those with increased expression in prostate cancer such as PCA3 and AGR2. Many studies have recently shown that urine PCA3 score could provide an additional tool for clinicians to manage their patients [31]. Bu *et al*. detected in urine AGR2 RNA by qRT-PCR involving 31 cases (positive biopsy) and 29 controls (negative biopsy) to show that urine AGR2 transcript outperformed serum PSA [32]. The nCounter CodeSet not only contains PCA3 and AGR2 but also probesets to other informative markers. Theoretically, the probe library could contain all genes (including ESTs) identified via gene expression analysis as up-regulated in prostate cancer cells.

The nCounter test is ideally suited for monitoring patients on active surveillance. These are men with a diagnosis of cancer but the pathology characteristics of their tumor would suggest a non-lethal cancer [33], [34]. An initial urine RNA signature for each patient is obtained at the time of enrollment. Each patient can then be monitored periodically by the nCounter test to determine if changes in the RNA signature correspond to disease progression as measured by PSA doubling time, Gleason grading, DRE, or evidence of cancer spread.

As a screening tool, all men could obtain a signature at age 40 and be followed annually or biannually for changes in this baseline urine RNA profile. A multiplex ELISA to measure prostate cancer secreted proteins (e.g., AGR2 and others) could eventually be developed to complement the RNA test. Thus, a correlation between AGR2 RNA counts and AGR2 protein amounts of the same specimens needs to be carried out. Urine supernatant is concentrated by spin filtration for measurement of AGR2 protein by the recently developed sandwich AGR2 ELISA [26]. Thus alternatively, all men could be screened by AGR2 ELISA. An AGR2 signal would then prompt nCounter testing to score the expression of other cancer markers for confirmation.

Lastly, the nCounter test is versatile and can incorporate markers for other urologic cancers such as bladder [30] and kidney. Furthermore, it is possible to include markers for any disease of the urinary tract organs to make it a multi-use clinical test. The collection and processing of urine is easy to perform and the cost is inexpensive (estimated at <$100 per test). More importantly, the digital data output from this test can be easily archived and tracked, and is easily understandable to doctors and patients.
